# Implementation of VA care coordination program to improve transitional care for veterans post-non-VA hospital discharge: an incremental cost analysis

**DOI:** 10.1186/s43058-023-00513-4

**Published:** 2023-11-13

**Authors:** Tiffany Radcliff, Roman Ayele, Marina McCreight, Wenhui Lui, Catherine Battaglia

**Affiliations:** 1 Denver-Seattle Center of Innovation, Department of Veterans Affairs, VA Eastern Colorado Healthcare System, MS 151, Aurora, CO USA; 2 School of Public Health, Texas A&M University, 157B SPH Administration Building, MS 1266, College Station, TX 77843-1266 USA; 3 Colorado School of Public Health, University of Colorado Anschutz Medical Campus, Aurora, CO USA

**Keywords:** Implementation science, Cost analysis, Care coordination, Veterans, Primary care, Nurses, Community care

## Abstract

**Background:**

The Veterans Affairs (VA) Healthcare System Community Hospital Transitions Program (CHTP) was implemented as a nurse-led intervention to reduce barriers that patients experience when transitioning from community hospitals to VA primary care settings. A previous analysis indicated that veterans who enrolled in CHTP received timely follow-up care and communications that improved care coordination, but did not examine cost implications for the VA.

**Methods:**

A budget impact analysis used the VA (payer) perspective. CHTP implementation team members and study records identified key resources required to initially implement and run the CHTP. Statistical analysis of program participants and matched controls at two study sites was used to estimate incremental VA primary care costs per veteran. Using combined program implementation, operations, and healthcare cost estimates to guide key model assumptions, overall CHTP costs were estimated for a 5-year time horizon, including a discount rate of 3%, annual inflation of 2.5%, and a sensitivity analysis that considered two options for staffing the program at VA Medical Center (VAMC) sites.

**Results:**

Implementation at two VAMCs required 3 months, including central program support and site-level onboarding, with costs of $34,094 (range: $25,355–$51,602), which included direct and indirect resource costs of personnel time, materials, space, and equipment. Subsequent annual costs to run the program at each site depended heavily on the staffing mix and caseload of veterans, with a baseline estimate of $193,802 to $264,868. Patients enrolled in CHTP had post-hospitalization VA primary care costs that were higher than matched controls. Over 5 years, CHTP sites staffed to serve 25–30 veterans per full-time equivalent transition team member per month had an estimated budget impact of $625 per veteran served if the transitional team included a medical social worker to support veterans with more social behavioral needs and less complex medical cases or $815 per veteran if nurses served all cases.

**Conclusions:**

Evidence-based care coordination programs that support patients’ return to VA primary care after a community hospital stay are feasible to implement and run. Further, flexibility in staffing this type of program is increasingly relevant as the VA and other healthcare systems consider methods to reduce provider burnout, optimize staffing, reduce costs, and address other staffing challenges while improving patient care.

**Supplementary Information:**

The online version contains supplementary material available at 10.1186/s43058-023-00513-4.

Contributions to the literature

- Cost analysis is an important component of implementation science, as it provides key guidance on the associated resources and financial implications of adopting evidence-based practices.
- There is growing evidence that supports the use of nurse-led transitional care programs after hospitalization, but there is little data on the costs of implementing and running these programs or the implications for primary care costs that are relevant for patients and payers.
- This study identifies cost implications of a nurse-only and nurse plus social worker staffing mix, recognizing that successful implementation includes optimization of staffing resources and patient needs.

## Background

Post-acute care coordination programs have been linked to better satisfaction with health care services, better health outcomes, and reduced duplication of services [1, 2]. Early interventions designed to improve care transitions focused on empowering patients and their caregivers to advocate for their needs during critical care transitions from hospitals to long-term care settings [3, 4], with newer programs focused on supporting better transitions of care from hospitals or emergency departments to primary care and other community-based healthcare settings [5–8]. Timely follow-up care in an ambulatory or primary care setting after a hospital stay is recognized as a best practice for medical homes [6, 9–17]. Further, this return to primary care has important economic ramifications for integrated delivery systems that both manage and finance patient care services. Poor coordination of care transitions for patients who access multiple systems of care, or who have multiple forms of health insurance, is a concern. For instance, an increasing number of American veterans use both Veterans Health Administration (VA) and non-VA systems of care and face challenges in navigating from non-VA community hospitals to VA-based primary care [18].

Efforts to improve access to healthcare in multiple systems for veterans include the VA’s Maintaining Internal Systems and Strengthening Integrated Outside Networks (MISSION) Act of 2018 [19, 20]. While this expansion of access was designed to increase access to timely care for veterans, it presented new healthcare coordination challenges and concerns that veterans that relied on the VA for their care might be at greater risk for adverse events when hospitalized in non-VA hospitals due to systems for transferring medical records, medications, and follow-up care that are incompatible. Several protocols for improving care transitions have been developed and implemented in the VA and have helped inform the evidence for developing best practices. Recent research reported that fragmented care could be improved with better transitions from non-VA hospitals to VA primary care [16]. Results included a finding that veterans who had helped coordinate their care had more primary care visits compared to a control group without support 120 days before their hospitalization, and no difference in 30-day hospital readmissions or emergency department visits and veterans who received care coordination support reported higher satisfaction with their care [16]. Another recent study reported that a transitional care program facilitated by nurses for rural veterans hospitalized in urban VA hospitals (i.e., the Rural Transitions Nurse Program) reduced both mortality and inpatient costs and increased outpatient primary care costs compared to controls [21, 22].

Strengthening care coordination through a nurse-led care transition program is a promising practice, but most studies that have tested the feasibility and described outcomes of such programs lacked details on resources and costs needed to inform implementation decisions. Growing evidence supports the importance of coordinated transitional care, but little is known about the cost of implementing and operating these programs, and there is limited information available on the budget impact of these programs for payers who are both coordinating and providing care [16]. To fill the gaps in identifying the value of care coordination for veterans who access multiple systems of care, this paper provides a descriptive cost analysis of the Community Hospital Transitions Program (CHTP), which was implemented in VA and shown to reduce barriers that patients experience when transitioning from community hospitals to VA primary care settings. This analysis identifies core features and related costs of CHTP. The type of economic information is needed both to help identify key resources for transition programs and inform the financial feasibility of similar programs designed to improve the patient experiences with care.

## Methods

The analysis described implementation, operating, and healthcare costs for a single care coordination program, CHTP, that was centrally housed within the Rocky Mountain Regional VA Medical Center (RMR VAMC) and implemented at two VAMC study sites. The analysis used the perspective of the VA as a payer for both the care transition program and VA-based primary care. No program was the implicit comparator for CHTP implementation (e.g., usual care). The study protocol was reviewed and approved as a Quality Improvement initiative by the Colorado Multiple Institutional Review Board (COMIRB protocol 15-1321), and the Veterans Health Services Research and Development (VA HSR&D) ethics review board.

### Program description

The protocol for CHTP has been described elsewhere [5, 16]. Briefly, veterans discharged from a non-VA community hospital to home or self-care between October 17, 2017, and July 10, 2020, were eligible to participate. Study enrollment in the last 4 months was lower than planned due to the onset of the COVID-19 pandemic, but the protocol was not changed. Patients referred by a community hospital for episodic transitional care were asked to verbally consent to participate in the program. Patients were eligible if they resided in Colorado or Nebraska, had previously established VA primary care at a VA site participating in the program, and were not already receiving case management services (e.g., support from an oncology social worker). Patients who were homeless, discharged to a setting other than the community (e.g., skilled nursing facilities), died during hospitalization, or had no discharge report (e.g., “observation only” patients) were excluded from the program. The program was managed centrally, but included transition teams that were housed at two individual sites. Individual sites had a 3-month implementation phase followed by an operational phase of at least 9 months.

Utilization and cost data from veterans who enrolled in CHTP were examined in comparison to propensity-matched controls to gage whether CHTP affected primary care utilization and costs. Detailed results of the statistical analysis (see Supplementary files 1 and 2) identified that CHTP cases were more likely to return to VA primary care and had higher post-hospitalization primary care costs compared to matched controls from the same VA location, and were used to guide assumptions (see Table 1) used in the cost analysis.

**Table 1:** Assumptions used for the CHTP cost analysis

	**Baseline estimate**	**Low range estimate**	**High range estimate**	**Data source/assumption notes**
**3-month program implementation staff time (hours)**				Study records, team interview for all staff effort estimates
Central program manager	112	100	168	Conducts training, implementation and supports all sites
Transitions nurse (RN)	40	40	60	At each VAMC site; trained by central program manager
Backup transitions nurse (RN)	20	-	30	At each VAMC site; trained by central program manager
Medical social worker (SW)	20	-	40	At 1 VAMC site; trained by central program manager
IT professional	50	50	75	Supports IVR/program IT needs (VA employee)
Site champion (MD Service Chief)	8	8	12	Single site, recruited by central program
**12-month program operations staff time (hours)**				Study records, team interviews
Central program manager	84	42	126	Central program support once implemented
Transitions nurse (RN)	2088	522	2088	1.0 FTE for Site 1, 0.25 FTE for site 2
Backup transitions nurse (RN)	522	209	1044	0.25 FTE (per site)
Medical social worker (SW)	0	0	1566	0.0 FTE for Site 1, 0.75 FTE for site 2
IT professional	16	16	32	Supports program IT needs (VA employee)
Site champion (MD Service Chief)	4	4	8	Provides as-needed support to transitions team
**Other program cost assumptions**				**Data source/assumption notes**
SQL training for program manager	$700	$700	$1400	1 time cost (implementation phase); study records, interview, high estimate assumes 2 trainings
Publicity materials for program	$3450	$1725	$6900	1 time (implementation phase), across sites, from study records and interview
Equipment (iPad and phone) for site	$2800	$2800	$3600	1 time cost (implementation phase), per site, study records
Travel Costs (in-person training)	$1257	$0	$2514	1 time cost (implementation phase), study records, baseline assumes 1 traveler per site, high estimate assumes 2
Medical records upload support	$200	$200	$400	1 time cost (implementation phase), team interview
Veteran contact cards, per case	$1.50			$1.50 per case, varies by number of cases per month
VA provided space and equipment	30%			VA overhead; assumed a fixed % of personnel costs
**Healthcare cost assumptions**				
Additional VA-based care	$96.30	$0.00	$ 160.00	Supplementary File and Supplementary Tables
**Program staff wages ($/hour)**	**Hourly wage**	**Hourly wage + 30% fringe**		https://www.bls.gov/oes/tables.htm
Central program manager	$29.80	$38.74		75th percentile from BLS table, 2021
Nurse (RN)	$46.91	$60.98		75th percentile from BLS table, 2021
Medical social worker (SW)	$36.98	$48.07		75th percentile from BLS table, 2021
IT professional	$38.57	$50.14		75th percentile from BLS table, 2021
Site champion (MD Service Chief)	$65.25	$84.83		MD Service Chief or Program Manager, 75th percentile from BLS table, 2021
**Discounting and inflation 5-year**				Used for 5-year Projections
Inflation rate, 2021 $ baseline year	2.5%			BLS medical inflation (average, 2018–2023, excluding 2020 due to COVID-19 anomalies)
Discount rate	3.0%			https://icer.org/wp-content/uploads/2020/10/ICER_2020_2023_VAF_102220.pdf

### Program costs

To implement CHTP, the study team followed the Quality Enhancement Research Initiative (QUERI) Roadmap to guide each phase of implementation [23]. Programmatic costs for the implementation phase were collected using a combination of semi-structured key informant interviews, study records, and assumptions that were initially developed by the study team before the implementation phase and later verified with key program personnel. The CHTP study protocol identified a preliminary list of resources and personnel that were required to start and manage the CHTP and established a process for program personnel to track and report their program-related time and caseload of patients each month. The preliminary list was revisited at the conclusion of the active program phase to guide semi-structured interviews with key program staff. Staff who were centrally managing the program at all sites and program staff at each site were separately asked to review the list and provide any adjustments that reflected the actual resources they used for implementation and operations at the two sites. The discussion included questions to identify specific programmatic costs that were new, variable, and specific to the care transition program. Further, program leaders and staff were asked to identify any areas where program costs varied from site to site or over time. Study documents and expense records were used to cross-confirm the recall of resources and related costs. The study team used a spreadsheet to track reported resources and related costs. Where ranges were reported, low and high estimates were used along with “typical” (baseline) reports to generate a low and high-cost estimate for each programmatic cost.

Personnel costs relied on the time logs to identify the time and effort to implement, train, and staff the program. Because a subset of staff initially engaged in both the implementation of the CHTP and its evaluation, the study team identified the proportion of time spent working on each and allocated the personnel costs accordingly and included a range of time estimates, where reports varied. To avoid complexities associated with local area wage differences across site, occupation categories (e.g., Registered Nurse) and experience levels of the program personnel were used in conjunction with publicly available national average hourly wage data from the US Bureau of Labor Statistics (BLS) National Occupational Employment and Wage Estimates (https://www.bls.gov/oes/tables.htm) for 2021 to calculate wage-based time costs. Actual hourly wages and levels of experience for VA personnel engaged in the program were similar to the 75th percentile of BLS-based national wage estimates, so the 75th percentile of the hourly wage for each occupational category identified for the program was used to calculate the baseline wage rates for personnel costs. A standard fringe benefit rate of 30% was included to account for additional non-salary costs of employee time. Further, costs of VA-provided space, support, and equipment were estimated as 30% of the direct personnel costs. All baseline programmatic costs were adjusted to constant 2021 dollars.

### Implementation costs

The initial list of program implementation resources included central personnel to develop program procedures for transitional teams to establish local community hospital partnerships for program referrals, professionally produced publicity materials for the program, training time and materials, onboarding and training transition teams for each site, travel, supplies and equipment, and indirect support (e.g., information technology and database development).

Following the literature that identifies best practices in considering costs in implementation studies, the costs of reallocated VA resources that were repurposed were included in the description of costs, while those that were otherwise unused (e.g., those without an opportunity cost) or that were on-time investments that would not be repeated for future expansion of the program to other sites were noted but not included as a cost for the program [24].

### Operating costs

To assess program site-level costs, study team members relied on site-level logs maintained by CHTP staff to track their time and caseloads while the program was operational. Program staff were asked to review resources identified from study records, including central program support and staffing and other items that were listed at the site level to maintain program operations. Key resources included a central program manager (across sites) to support training and trouble-shooting, a dedicated transition staff of at least one full-time equivalent (1.0 FTE), a part-time backup transition nurse for each site, and ongoing access to a site Champion (generally an MD, Service Chief or Program Director) to assure local support for the program, and general IT and other VA support resources needed to maintain consistent services to patients who enrolled in the program. Because the primary objective of the cost analysis was to identify program feasibility for sustained operations, initial operating costs at each site were estimated to reflect a full 12 months of program operations, regardless of how long the site was operational during the CHTP study. Further, program leaders and staff were asked to identify the expected caseload of veterans that could be identified and served per month once a site was fully operational and how transition team staffing levels could be scaled to match anticipated increased demand for the program over time.

### Healthcare costs

Incremental VA-based primary care costs were included in the analysis because the intervention was designed to facilitate the return of veterans to VA-based primary care after a community hospital stay. Primary care cost estimate assumptions relied on a detailed statistical analysis of main CHTP outcomes that compared veterans who received the intervention to a propensity-matched sample of controls at the same VA sites who did not access CHTP (e.g., usual care). While both groups had an increase in primary care visits within 120 days following the index hospitalization, a difference-in-differences analysis indicated that CHTP cases had a 14.4% larger increase compared to controls (95% CI = 2.5 to 27.6%, *p* = 0.02). Supplementary file 1 includes full details on the main outcome analysis and matching methods. Briefly, matching was conducted using the “Matchit” algorithm in the R statistical package, with an exact match on the CHTP site and nearest neighbor matching for other variables [25]. Due to the large sample of veterans available to match, the study used a 2:1 matching ratio of controls to CHTP participants. The analytic steps required to match intervention cases to controls and analysis of VA-provided primary care costs were conducted in R Version 4.0.3 [26]. Control sample cases had a higher probability of death within 30 days (2.6% versus 0.8%,* p* < 0.005) and 60 days (3.4% versus 1.8%, *p* < 0.039), but no differences in the probability of death within 90 days and beyond (4.3% versus 2.7%, *p* < 0.071) and post-match patient-level characteristics that were statistically similar (see Supplementary File 1).

VA primary care costs for CHTP cases and controls were identified in the VA’s Managerial Cost Accounting (MCA) files, which used activity-based costs for encounters within the VA, including primary care encounters and other clinic visits. MCA captured both direct costs (e.g., personnel salaries, medical supplies) and indirect costs (e.g., overhead expenses) associated with providing care. R software was used to assess pre-parallel trends in costs, and then MS Excel® software was used to calculate descriptive differences in median primary care costs. Differences in median costs were selected to guide the baseline analysis because of influential outliers identified in the mean cost estimates and relied on a 120-day window before and after the index hospitalization; this 120-day window was used for all primary CHTP outcomes.

### Estimated program implementation, delivery, and healthcare costs

Using assumptions for implementation phases listed in Table 1, MS Excel® software was used to calculate the following: (1) initial 3-month CHTP implementation costs (Table 2), (2) 12-month operating costs using the staffing reported for each CHTP site (Table 3), and (3) estimated 5-year budget implications of CHTP for the VA, including incremental healthcare costs (Table 4). The assumptions 5-year estimates used constant 2021 dollars for the first year (implementation and 9 months of operations) and included a 2.5% medical inflation rate for subsequent years. A discount rate of 3% was used in the 5-year cost projections. Additional sensitivity checks included sensitivity analysis which included the median incremental difference in all VA outpatient costs (versus primary care) during the window and in-person versus online training for program personnel, noting that travel for training was planned and used prior to the onset of the COVID-19 pandemic in March 2020, but was replaced with remote video conferencing.

**Table 2:** Three-month CHTP implementation costs

	**Baseline**	**Low estimate**	**High estimate**
*Central program (covers both sites)*			
Program manager time (salary and fringe)	$4339	$3874	$6508
SQL training course tuition	$700	$700	$1400
Publicity materials for program (contracted)	$3450	$1725	$6900
Travel costs (for in-person training)	$1257	$ -	$2514
Re-allocated Resources			
HIMS support (medical records upload)	$200	$200	$400
IT programming/IVR support	$2507	$2507	$3761
VA provided space and equipment	$2114	$1974	$3201
Total: central program	$14,567	$10,980	$24,684
*VAMC Site 1—nurse-only transitions team*			
Transition nurse (salary and fringe)	$2439	$2439	$3659
Backup transitions nurse (salary and fringe)	$1220	$ -	$1829
Equipment (iPad and phone) for transitions team	$2800	$2800	$3600
Re-allocated resources			
Site champion	$679	$679	$1018
VA provided space and equipment	$1301	$935	$1952
Total: Site 1	$8439	$6853	$12,058
*VAMC Site 2—nurse/social worker transitions team*			
Transition nurse (salary and fringe)	$2439	$2,439	$3659
Backup transition nurse (salary and fringe)	$1220	$ -	$1829
Healthcare social worker (salary and fringe)	$961	$ -	$1923
Equipment (iPad and phone) for transitions team	$4200	$4200	$5000
Re-allocated resources			
Site champion (salary and fringe)	$679	$679	$1018
VA provided space and equipment	$1590	$204	$1431
Total: Site 2	$11,089	$7522	$14,860
***Total implementation costs (all sites)***	***$34,094***	***$25,355***	***$51,602***
Total implementation costs (Central and Site 1)	$23,006	$17,834	$36,742
Total Implementation costs (Central and Site 2)	$25,655	$18,502	$39,544

**Table 3:** Twelve-month CHTP delivery costs for VA sites serving 25–30 cases per month

	**VA Site 1 (1.0 FTE RN only)**			**VA Site 2 (0.25 FTE RN +0.75 FTE SW)**		
	**Baseline**	**Low estimate**	**High estimate**	**Baseline**	**Low estimate**	**High estimate**
*Personnel time and effort (salary + fringe)*						
Central program manager (serves all sites)	$3254	$1627	$4881	$3254	$1627	$4881
Transitions staff^a^ (1.0 FTE, RN, or RN+SW)	$167,170	$100,302	$200,604	$112,504	$84,378	$168,755
Backup transitions nurse (0.25 FTE)	$31,833	$12,733	$63,666	$31,833	$12,733	$63,666
*Re-allocated personnel resources*						
IT programming/IVR support	$802	$802	$1605	$802	$802	$1605
Site champion	$339	$339	$679	$339	$339	$679
Total personnel	$203,398	$115,804	$271,434	$148,732	$99,880	$239,586
*Materials and other costs*						
Postage and mailings for veteran care cards	$450	$400	$500	$450	$450	$540
VA provided space, equipment, overhead	$61,020	$34,741	$81,430	$44,620	$29,964	$71,876
Total materials, overhead, and other	$61,470	$35,141	$81,930	$45,070	$30,414	$72,416
**Total annual operating costs**	$264,868	$150,945	$353,364	$193,802	$130,294	$312,002

^a^Registered nurse (RN)-only at Site 1, blended RN and medical social worker (SW) at Site 2

**Table 4:** Projected 5-year CHTP costs to VA

**Program year**	**Start up (3 months)**	**1 (9 months)**	**2 (12 months)**	**3 (12 months)**	**4 (12 months)**	**5 (12months)**	**5-year overall total**^d^	**Average per veteran**^e^
Veterans served ^a^	-	225	300	720	720	720	2685	
Transition team (FTE)^a^		0.75	1.0	2.0	2.0	2.0		
**VA investment**^b^								
Central Program Support	$14,567	$4881	$6671	$6838	$6968	$7184	$42,951	$16.00
VAMC Site 1 (RN-only)	$8439	$196,210	$268,154	$549,716	$563,459	$577,546	$1,896,685	$706.40
VAMC Site 2 (RN/SW)	$11,089	$142,911	$195,312	$400,389	$410,399	$420,658	$1,386,259	$516.30
Total program costs (Central and Site 1 only)	$23,006	$201,092	$274,825	$556,554	$570,427	$584,730	$1,939,636	$722.40
Total program costs (Central and Site 2 only)	$25,655	$147,792	$201,983	$407,227	$417,366	$427,842	$1,429,210	$532.29
Total program costs (Central and both sites)	$34,094	$344,003	$470,137	$956,943	$980,825	$1,005,388	$3,325,895	$619.35
**Healthcare Costs**^b,c^								
VA primary care (per site)		$21,668	$29,612	$72,846	$74,667	$76,534	$247,973	$92.35
**Total Costs -- Program Delivery and Healthcare**^b^								
Central and Site 1 only	$23,006	$222,759	$304,438	$629,400	$645,094	$661,263	$2,187,609	$814.75
Central and Site 2 only	$25,655	$169,460	$231,595	$480,073	$492,034	$504,376	$1,677,183	$624.65
Central and both sites	$34,094	$365,670	$499,749	$1,029,789	$1055,493	$1,081,922	$3,573,868	$665.52

^a^Program is assumed to increase staffing to 2.0 FTE by year 3 and increase caseloads from 25 to 30 cases per FTE per month

^b^Adjusted to constant 2021 dollars for Start-up/Year 1, assumes 2.5% annual inflation in subsequent years

^c^Incremental VA primary care costs per site

^d^5-year totals were discounted by 3%

^e^5-year overall total divided by veterans served over 5 years

## Results

### Implementation costs

Table 2 summarizes the costs associated with CHTP implementation. Implementation costs per site included personnel to initiate the program and provide training, including a program manager, an experienced nurse to lead the program, network/IT support for programming and other technical support, and a site champion. Start-up costs also included personnel time dedicated to onboarding and training, though the central program manager’s efforts were more extensive, and involved arranging for training, equipment set-up, learning new software, and coordinating the work of site personnel to ensure a strong start. In addition, due to uncertainty in the continued availability of a single transition nurse, participating sites each identified and trained a backup transition nurse to ensure the continuity of the program. As implemented, travel costs were incurred for face-to-face training prior to the COVID-19 pandemic and are reported in the baseline and high-range estimate. Materials costs included an Apple iPad and mobile phone service for all members of the transition team at each site, publicity materials to notify veterans and their community hospital providers of the program, modest support for medical records uploading, and VA-provided space for the transition team and central program staff to work. The estimated implementation costs for both sites totaled $34,094 (range: $25,355–$51,602), including central program support. Site 1 relied on nurses (RNs) only to deliver the program, while Site 2 included a medical social worker (SW) in addition to the nurse, which increased site-level implementation costs by about $2650 due to the added personnel time and equipment costs required for training. Low-range estimates of implementation costs of $17,834 were for a single site with central support, but excluded the backup transition nurse, social worker, and eliminated travel for site-level training (e.g., assumed training would use VA-approved online meeting capabilities such as MS Teams). The higher-range estimated implementation costs added more personnel hours for training and onboarding sites, additional equipment for transition teams, and higher travel costs for in-person training.

### Annual operating costs

Table 3 provides a summary of the estimated annual (12-month) program delivery costs for each CHTP VAMC site staffed to support 25 transitional care cases per month with either a single full-time equivalent (1.0 FTE) transition nurse (Site 1) or a nurse/social worker team (0.25 FTE nurse and 0.75 FTE social worker) for transitional care (Site 2), along with a backup transition nurse to ensure program continuity for the site. Primary cost differences between sites are due to the wage differential for nurses versus social workers, with $71,066 in annual baseline cost differences for the two sites. Site 2, which used the blended RN+SW model, had estimated annual operating costs of $193,802 per year and the RN-only model used by Site 1 was about $264,868 per year. Sensitivity analyses varied the effort of the central program manager (from 42 to 126 h per year), backup transition nurse (from 0.10 FTE to 0.5 FTE), and related costs of space, support, and equipment, with a resulting range of $107,435–$353,364 in annual operating cost estimated from (see Table 3).

Table 4 provides the estimated 5-year budget impact of the program. These estimates assumed that (1) each site would be fully operational by month 4 of the initial year and (2) caseloads per full-time equivalent transition team would increase from 25 to 30 cases per site team member per month and an additional 1.0 FTE would be added to the CHTP transition team (from 1.0 FTE to 2.0 FTE) in year 3 due to a mixture of increased team experience and a greater volume of referrals by community hospitals. With two sites staffed to serve up to 2685 cumulative cases over 57 months of active operations, summed implementation and operating costs range from $532 per veteran if nurses support only the most complex cases and social workers support other cases (e.g., the Site 2 staffing model is implemented), to $722 per veteran for an entirely nurse-led and staffed program (e.g., the Site 1 staffing model is implemented). Incremental primary care costs, with a discounting of 3%, were estimated to increase VA care costs per site by $247,973 per year, or about $92 per veteran served. The upper-end estimated increase in healthcare costs which included median differences in all outpatient costs (vs. incremental primary care costs) was $412,001 per site, or $153.50 per veteran served (data not shown).

## Discussion

The CHTP was implemented at two VA medical centers to test whether a nurse-led program would be an effective way to facilitate transitional care including the timely return of veteran patients to VA primary care after a community hospital stay. The results of this cost analysis indicate that the CHTP had modest implementation costs; implementation primarily required training key personnel to manage the program at each site and providing the site and study team with technology to support care coordination. Initial 12-month operating costs were estimated at $264,868 for a site with a single transition nurse handling a caseload of 25 transitions per month or $193,802 if the staffing was modified from a nurse-only model to include a social worker to support veterans with more social behavioral needs and less medically complex transitions of care. Beyond the initial implementation and year of operations, moderate economies of scale would be expected; lower costs per case would be expected due to expanded caseloads for transition team and more staffing capacity to manage cases. The CHTP, as implemented, effectively leveraged existing VA resources that were relevant to the intervention (e.g., IT support, space, and other fixed resources). These types of resources may be important to consider for assessing space, staffing, and other investment decisions for individual medical centers considering care transition programs.

It is difficult to compare the costs of CHTP with other care coordination programs since program components, assessments, and target populations widely differ. The VA Rural Transitions Nurse Program identified larger increases in outpatient medical costs for cases versus controls, but did not measure program implementation and operational costs [21]. VA’s Coordinated Transitional Care (C-TraC) program recipients had one-third fewer rehospitalizations than those in a baseline comparison group, producing an estimated savings of $1225 per patient net of programmatic costs. The implementation of this low-cost transitional care program significantly reduced hospital readmissions [27].

This study provides a descriptive analysis of the cost implications of the CHTP for VA and has some important limitations. Among these is the challenge that was encountered in enrolling patients for the intervention during the early phases of the COVID-19 pandemic; the veterans who received the intervention during this time may not be representative of all veterans who would normally seek community hospitalizations. Estimates in this paper are based on assumptions that the model is scalable. Additional studies should be conducted to assess the scalability of the intervention, including the impact of staffing the program with transition nurse to manage complex cases and other professionals (e.g., social workers) to support more straightforward cases. The assessment of VA-based primary care and other outpatient costs is a single indicator of program outcomes and does not capture other care that may have been sought outside of VA by either CHTP cases or controls and does not capture the broader societal perspective. Future work to identify and capture broader costs and cost savings of care transition programs is needed. In addition, the cases and sites studied to identify healthcare cost parameters are not generalizable for future implementation planning. However, the methods to identify descriptive differences between cases and matched controls adjusted for many of the challenges inherent in observational studies of care utilization costs before and after an intervention. The descriptive finding that primary care costs increased more for intervention cases compared to matched controls suggests that the CHTP, as implemented, was successful in encouraging reintegration of veterans with VA-based primary care.

## Conclusion

The CHTP presents a promising practice to consider for broader adoption. This research identified that the program required modest investments by the VA to implement and operate at each site and required limited VA staffing and other resources to sustain the program once established. Implementation of the program was supported by a growing evidence base that the provision of effective transitions to primary care following hospitalization is vital for establishing a patient-centered medical home. Further, the assessment of program delivery options and related cost implications is increasingly relevant as the VA and other healthcare systems consider methods to reduce provider burnout and other staffing challenges while improving the patient experience during care transitions.

### Supplementary Information

**Additional file 1.** Matching details.

**Additional file 2:** **Supplementary Tables.** Assessment of Healthcare Cost Differences. **Table 1.** VA primary care costs 120 Days before and after index hospitalization. **Table 2.** VA outpatient care costs 120 Days before and after index hospitalization. **Figure 1.** Pre-parallel trends in 120-day non-primary care costs (including zero costs). **Figure 2.** Pre-parallel trends in 120-day primary care costs (including zero costs).

## Data Availability

The patient-level data that supports the findings of this study are available from the US Department of Veterans Affairs but restrictions apply to the availability of these data beyond the current study. Data are available from the authors upon reasonable request and with the permission of the US Department of Veterans Affairs
