# Supplementary material for: Implementation of VA care coordination program to improve transitional care for veterans post-non-VA hospital discharge: an incremental cost analysis
Source: Implement Sci Commun. 2023 Nov 13;4:135. doi: 10.1186/s43058-023-00513-4 (PMC10642017; doi:10.1186/s43058-023-00513-4)
Supplement: Supplementary file 1 — Additional file 1. Matching details. [file 43058_2023_513_MOESM1_ESM.docx]

**Community Hospital Transitions Program (CHTP) Supplementary Details**

Cohort Description

The CHTP intervention group were patients who had community hospital inpatient stay and were eligible and enrolled in CHTP from 10/17/2017 to 7/10/2020 and discharged to home/self-care. A patient could repeatedly enroll into CHTP program at different hospitalization, we only included their index enrollment in the analyses, to avoid the tangle of treatment effect and number of treatment additive effect to bias the result. Using the VA Corporate Data Warehouse (CDW) Fee and PIT data, control group were selected from veterans who discharged from community hospitals to home/self-care, from the same time period as above, from the same site VA medical centers as CHTP intervention patients. We also only use their index hospitalization within this time frame for the control group.

Data Sources

The CDW Fee Basis files contain data for care received through non-VA care program, including the Choice Act, It’s the primary source of VA Community Care data before the onset of PIT. Beginning in 2013, VA introduced the Non-VA Care Program Integrity Tools (PIT) system, which is a comprehensive set of tools that aggregates many sources of data to check for fraud, waste, and abuse in the VA Community Care program. PIT data include multiple claims data sources and have comprehensive data on all Veteran family member programs, and Choice and MISSION Act utilization. Due to gaps between data sources, we need use both CDW Fee basis and PIT data to identify the control group for CHTP study. VA CDW is a centralized database that consolidates data from various sources within the VA healthcare system, including the VistA electronic health record, financial systems, pharmacy systems, laboratory systems, and others. It provides a comprehensive view of patient information, diagnoses, procedures, medications, and other healthcare-related data within the VA system. The Non-VA Care Program Integrity Tools (PIT) system is a comprehensive set of tools that VA used during the study timeframe to aggregate many sources of data in the VA Community Care program to identify potential fraud, waste, and abuse. PIT data include multiple claims data sources and have comprehensive data on all Veteran family member programs, and Choice and MISSION Act utilization.

For our primary analyses, the intervention group only included patients who completed all four components of the CHTP intervention. 124 eligible CHTP patients died before they had chance to complete all 4 core components, we had to exclude them from the primary analysis. An intent-to-treat analysis was performed as sensitivity analysis by including all eligible CHTP patients as intervention group, no matter how many core components they finished.

There were 1164 eligible CHTP patients of which 774 completed all four components. There were 7830 eligible control patients pulled from CDW data.

Primary Outcome

The primary outcome of interest was the return to the VA primary care (the veterans medical home) following discharge from a community (non-VA) hospitalization.

Matching

Below is the Table 1 to compare patient characteristics as well as 1-year pre and post intervention between Control and CHTP patients prior to matching. It shows all the patient factors and most of the Elixhauser comorbidity index variables were significantly different before matching. Matching used the “Match-It” package. (Ho et al., 2011)

Table 1: Comparison of control and CHTP group Pre-Matching

|  |  | **Control** | **CHTP** | **p-value** |  |
| --- | --- | --- | --- | --- | --- |
|  |  | (N=7830) | (N=774) |  |  |
| **Patient Factors** |  |  |  |  |  |
| age (mean (SD)) |  | 58.20 (17.60) | 67.47 (11.50) | <0.001 |  |
| gender = F (%) |  | 1180 (15.1) | 51 ( 6.6) | <0.001 |  |
| race4 (%) |  |  |  | <0.001 |  |
| White |  | 6428 (82.1) | 621 (80.2) |  |  |
| Black |  | 632 ( 8.1) | 102 (13.2) |  |  |
| Other |  | 244 ( 3.1) | 19 ( 2.5) |  |  |
| Unknown |  | 526 ( 6.7) | 32 ( 4.1) |  |  |
| URH (%) |  |  |  | <0.001 |  |
| Urban |  | 4917 (62.8) | 596 (77.0) |  |  |
| Rural |  | 2657 (33.9) | 172 (22.2) |  |  |
| Highly Rural |  | 256 ( 3.3) | 6 ( 0.8) |  |  |
| Site (%) |  |  |  | <0.001 |  |
| Omaha |  | 3421 (43.7) | 237 (30.6) |  |  |
| Denver |  | 4409 (56.3) | 537 (69.4) |  |  |
| **Elixhauser Comorbidity Index** |  |  |  |  |  |
| HTN_C = 1 (%) |  | 5243 (67.0) | 607 (78.4) | <0.001 |  |
| CHF = 1 (%) |  | 1528 (19.5) | 228 (29.5) | <0.001 |  |
| CHRNLUNG = 1 (%) |  | 2742 (35.0) | 314 (40.6) | 0.002 |  |
| DM = 1 (%) |  | 1930 (24.6) | 290 (37.5) | <0.001 |  |
| DMCX = 1 (%) |  | 1757 (22.4) | 275 (35.5) | <0.001 |  |
| RENLFAIL = 1 (%) |  | 1350 (17.2) | 193 (24.9) | <0.001 |  |
| OBESE = 1 (%) |  | 2162 (27.6) | 193 (24.9) | 0.121 |  |
| WGHTLOSS = 1 (%) |  | 732 ( 9.3) | 87 (11.2) | 0.100 |  |
| LYTES = 1 (%) |  | 2907 (37.1) | 338 (43.7) | <0.001 |  |
| AIDS = 1 (%) |  | 29 ( 0.4) | 3 ( 0.4) | 1.000 |  |
| ALCOHOL = 1 (%) |  | 1574 (20.1) | 109 (14.1) | <0.001 |  |
| ANEMDEF = 1 (%) |  | 1551 (19.8) | 236 (30.5) | <0.001 |  |
| ARTH = 1 (%) |  | 444 ( 5.7) | 53 ( 6.8) | 0.208 |  |
| BLDLOSS = 1 (%) |  | 231 ( 3.0) | 19 ( 2.5) | 0.502 |  |
| COAG = 1 (%) |  | 792 (10.1) | 119 (15.4) | <0.001 |  |
| DEPRESS = 1 (%) |  | 2904 (37.1) | 220 (28.4) | <0.001 |  |
| DRUG = 1 (%) |  | 1112 (14.2) | 68 ( 8.8) | <0.001 |  |
| HYPOTHY = 1 (%) |  | 997 (12.7) | 112 (14.5) | 0.187 |  |
| LIVER = 1 (%) |  | 1103 (14.1) | 128 (16.5) | 0.071 |  |
| LYMPH = 1 (%) |  | 111 ( 1.4) | 14 ( 1.8) | 0.478 |  |
| METS = 1 (%) |  | 323 ( 4.1) | 22 ( 2.8) | 0.101 |  |
| NEURO = 1 (%) |  | 2074 (26.5) | 223 (28.8) | 0.177 |  |
| PARA = 1 (%) |  | 310 ( 4.0) | 39 ( 5.0) | 0.175 |  |
| PERIVASC = 1 (%) |  | 1438 (18.4) | 218 (28.2) | <0.001 |  |
| PSYCH = 1 (%) |  | 1295 (16.5) | 67 ( 8.7) | <0.001 |  |
| PULMCIRC = 1 (%) |  | 393 ( 5.0) | 53 ( 6.8) | 0.035 |  |
| TUMOR = 1 (%) |  | 805 (10.3) | 83 (10.7) | 0.746 |  |
| ULCER = 1 (%) |  | 231 ( 3.0) | 32 ( 4.1) | 0.086 |  |
| VALVE = 1 (%) |  | 1117 (14.3) | 179 (23.1) | <0.001 |  |
| **prior Intervention** |  |  |  |  |  |
| 1yr Prior PCP visit (mean (SD)) |  | 1.70 (2.64) | 1.11 (2.15) | <0.001 |  |
| 1yr Prior hospitalization (mean (SD)) |  | 0.59 (1.22) | 0.66 (1.27) | 0.189 |  |
| 1yr Prior ED Visit (mean (SD)) |  | 1.71 (3.11) | 2.16 (3.37) | <0.001 |  |

The name of the Elixhauser comorbidity index variable is listed below, corresponding to their abbreviation in table above.

AIDS = AIDS/HIV

ALCOHOL = Alcohol Abuse

ANEMDEF = Anemia Defficiency

ARTH = Rheumatoid Arthritis

BLDLOSS = Blood Loss Anemia

CARDARRH = Cardiac Arrhythmia

CHF = Congestive Heart Failure

CHRNLUNG = Chronic Pulmonary Disease

COAG = Coagulopathy

DEPRESS = Depression

DM = Diabetes without Chronic Complications

DMCX = Diabetes with Chronic Complications

DRUG = Drug Abuse

HTN_C = Hypertension

HYPOTHY = Hypothyroidism

LIVER = Liver Disease

LYMPH = Lymphoma

LYTES = Fluid and Electrolyte Disorders

METS = Metastatic Cancer

NEURO = Other Neurological Disorders

OBESE = Obesity

PARA = Paralysis

PERIVASC = Peripheral Vascular Disease

Below are figures of propensity score before and after matching. There was one CHTP patient was removed from the cohort due to the aspects that highly differed from the control group identifying no suitable match existed (figure 1). The histogram plot compared the propensity score distribution between CHTP and control group before and after matching. It showed there was big improvement for control group after matching (figure 2).

Figure 1. Propensity score by matched and unmatched per control and CHTP group.


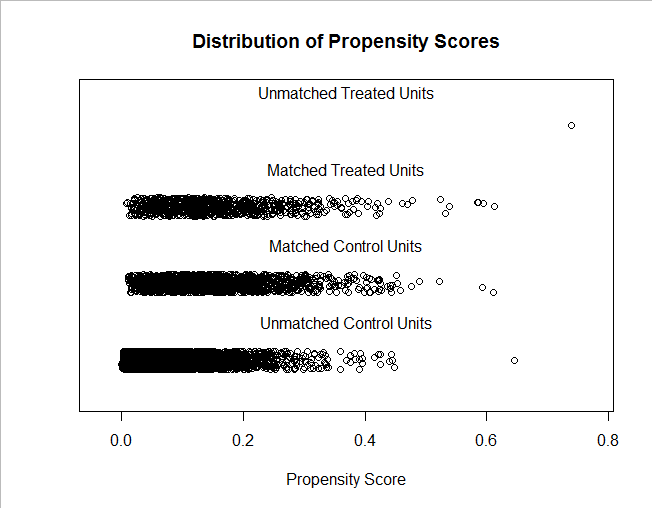


Figure 2. Histogram of propensity score by CHTP and control group before and after matching.


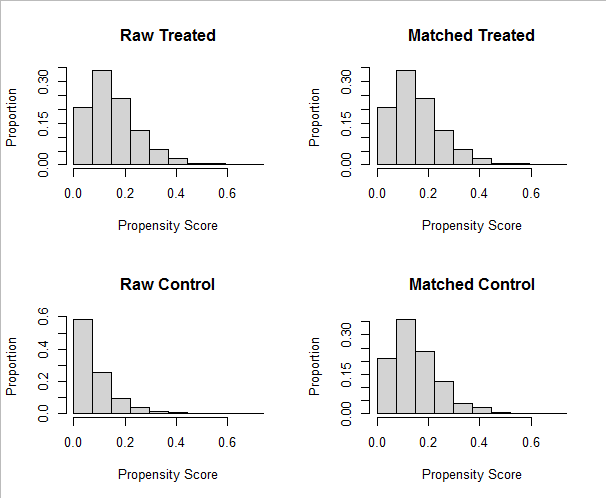


Table 2 below is the standardized difference of CHTP and control group before and after matching. Standardized differences less than 0.1(10%) between control and treatment groups are commonly considered as negligible imbalance. After matching, all standardized differences between CHTP and control groups were below .05 for predictive covariates, indicating appropriate covariate balance.

Table 2: Standardized mean difference on matched variables before and after matching

|  | **Std. Mean Diff. before matching** | **Std. Mean Diff. after matching** |
| --- | --- | --- |
| distance | 0.7359121 | 0.0626034 |
| Age | 0.8063521 | -0.0139912 |
| GenderM | 0.3418524 | 0.0260722 |
| URHU | 0.3375739 | 0.0000000 |
| URHR | -0.2816995 | 0.0000000 |
| URHH | -0.2844005 | 0.0000000 |
| Site | -0.2834003 | 0.0000000 |
| race4 White | -0.0467539 | 0.0048726 |
| race4 Black | 0.1509743 | 0.0019123 |
| race4 Other | -0.0427445 | 0.0083601 |
| race4 Unknown | -0.1297637 | -0.0194942 |
| HTN_C1 | 0.2786766 | 0.0000000 |
| CHF1 | 0.2181125 | 0.0297980 |
| CHRNLUNG1 | 0.1130152 | 0.0000000 |
| DM1 | 0.2648320 | 0.0146995 |
| DMCX1 | 0.2735121 | 0.0473024 |
| RENLFAIL1 | 0.1778393 | -0.0029902 |
| OBESE1 | -0.0618610 | -0.0119606 |
| WGHTLOSS1 | 0.0598886 | -0.0245739 |
| LYTES1 | 0.1319180 | 0.0039125 |
| AIDS1 | 0.0027724 | 0.0000000 |
| ALCOHOL1 | -0.1730518 | -0.0260337 |
| ANEMDEF1 | 0.2320427 | 0.0028101 |
| ARTH1 | 0.0466046 | -0.0179277 |
| BLDLOSS1 | -0.0320152 | 0.0125401 |
| COAG1 | 0.1458178 | 0.0179324 |
| DEPRESS1 | -0.1920926 | -0.0458897 |
| DRUG1 | -0.1913302 | -0.0388440 |
| HYPOTHY1 | 0.0493804 | -0.0165476 |
| LIVER1 | 0.0659623 | 0.0034821 |
| LYMPH1 | 0.0293512 | -0.0145607 |
| METS1 | -0.0771922 | -0.0077847 |
| NEURO1 | 0.0513045 | 0.0000000 |
| PARA1 | 0.0493559 | -0.0059141 |
| PERIVASC1 | 0.2178745 | 0.0359506 |
| PSYCH1 | -0.2803268 | 0.0000000 |
| PULMCIRC1 | 0.0723942 | -0.0076833 |
| TUMOR1 | 0.0143027 | 0.0062715 |
| ULCER1 | 0.0594812 | 0.0000000 |
| VALVE1 | 0.2101538 | 0.0138067 |
| 1yr Prior ED Visit | 0.1222412 | 0.0409671 |
| 1yr Prior Hospitalization | 0.0475208 | 0.0264619 |
| 1yr Prior PCP Visit | -0.2758923 | -0.0333830 |

Below is Table 3, the data after matching, comparing the CHTP and control groups on patient factors, comorbidity index variables, 1-year outcomes prior discharge, and unadjusted outcomes post discharge. We use chi-square tests to compare categorical data and Mann-Whitney Wilcoxon nonparametric tests for continuous data. After matching, there were no significant differences between CHTP and control groups in patient factor, comorbidity variables, and 1-year prior ED visit, admission, and PCP visit.

The unadjusted outcome rates between the two groups were also not significantly different for most of them. The control group had significantly higher unadjusted 30-day and 60-day mortality rates than CHTP group.

Table 3: Comparison of control and CHTP group post-Matching.

|  | **Control** | **CHTP** | **p-value** |  |
| --- | --- | --- | --- | --- |
|  | (N=1546) | (N=773) |  |  |
| **Patient Factors** |  |  |  |  |
| age (mean (SD)) | 67.60 (12.63) | 67.44 (11.47) | 0.766 |  |
| gender = F (%) | 112 ( 7.2) | 51 ( 6.6) | 0.625 |  |
| race4 (%) |  |  | 0.975 |  |
| White | 1239 (80.1) | 621 (80.3) |  |  |
| Black | 201 (13.0) | 101 (13.1) |  |  |
| Other | 36 ( 2.3) | 19 ( 2.5) |  |  |
| Unknown | 70 ( 4.5) | 32 ( 4.1) |  |  |
| URH (%) |  |  | 1.000 |  |
| U | 1190 (77.0) | 595 (77.0) |  |  |
| R | 344 (22.3) | 172 (22.3) |  |  |
| H | 12 ( 0.8) | 6 ( 0.8) |  |  |
| Site (%) |  |  | 1.000 |  |
| Omaha | 472 (30.5) | 236 (30.5) |  |  |
| Denver | 1074 (69.5) | 537 (69.5) |  |  |
| **Elixhauser Comorbidity Index** |  |  |  |  |
| HTN_C = 1 (%) | 1212 (78.4) | 606 (78.4) | 1.000 |  |
| CHF = 1 (%) | 433 (28.0) | 227 (29.4) | 0.526 |  |
| CHRNLUNG = 1 (%) | 626 (40.5) | 313 (40.5) | 1.000 |  |
| DM = 1 (%) | 567 (36.7) | 289 (37.4) | 0.773 |  |
| DMCX = 1 (%) | 513 (33.2) | 274 (35.4) | 0.299 |  |
| RENLFAIL = 1 (%) | 386 (25.0) | 192 (24.8) | 0.986 |  |
| OBESE = 1 (%) | 394 (25.5) | 193 (25.0) | 0.826 |  |
| WGHTLOSS = 1 (%) | 184 (11.9) | 86 (11.1) | 0.631 |  |
| LYTES = 1 (%) | 671 (43.4) | 337 (43.6) | 0.965 |  |
| AIDS = 1 (%) | 6 ( 0.4) | 3 ( 0.4) | 1.000 |  |
| ALCOHOL = 1 (%) | 232 (15.0) | 109 (14.1) | 0.604 |  |
| ANEMDEF = 1 (%) | 468 (30.3) | 235 (30.4) | 0.987 |  |
| ARTH = 1 (%) | 113 ( 7.3) | 53 ( 6.9) | 0.754 |  |
| BLDLOSS = 1 (%) | 35 ( 2.3) | 19 ( 2.5) | 0.884 |  |
| COAG = 1 (%) | 226 (14.6) | 118 (15.3) | 0.725 |  |
| DEPRESS = 1 (%) | 472 (30.5) | 220 (28.5) | 0.328 |  |
| DRUG = 1 (%) | 153 ( 9.9) | 68 ( 8.8) | 0.438 |  |
| HYPOTHY = 1 (%) | 233 (15.1) | 112 (14.5) | 0.757 |  |
| LIVER = 1 (%) | 254 (16.4) | 128 (16.6) | 0.984 |  |
| LYMPH = 1 (%) | 31 ( 2.0) | 14 ( 1.8) | 0.873 |  |
| METS = 1 (%) | 46 ( 3.0) | 22 ( 2.8) | 0.965 |  |
| NEURO = 1 (%) | 446 (28.8) | 223 (28.8) | 1.000 |  |
| PARA = 1 (%) | 80 ( 5.2) | 39 ( 5.0) | 0.973 |  |
| PERIVASC = 1 (%) | 409 (26.5) | 217 (28.1) | 0.437 |  |
| PSYCH = 1 (%) | 134 ( 8.7) | 67 ( 8.7) | 1.000 |  |
| PULMCIRC = 1 (%) | 109 ( 7.1) | 53 ( 6.9) | 0.931 |  |
| TUMOR = 1 (%) | 161 (10.4) | 82 (10.6) | 0.943 |  |
| ULCER = 1 (%) | 64 ( 4.1) | 32 ( 4.1) | 1.000 |  |
| VALVE = 1 (%) | 349 (22.6) | 179 (23.2) | 0.793 |  |
| **Prior Intervention** |  |  |  |  |
| 1yr Prior PCP visit (mean (SD)) | 1.18 (1.93) | 1.11 (2.15) | 0.416 |  |
| 1yr Prior hospitalization (mean (SD)) | 0.62 (1.20) | 0.65 (1.26) | 0.532 |  |
| 1yr Prior ED Visit (mean (SD)) | 1.99 (3.58) | 2.14 (3.68) | 0.338 |  |

Difference-in-Difference

Difference-in-Difference is defined as “the difference in average outcome in the treatment group before and after treatment minus the difference in average outcome in the control group before and after treatment”. Assuming the trend of outcomes over time would be the same for matched control and intervention groups if the intervention had never occurred, if there is difference observed in pre-post trend between the two groups, then the difference is caused by the intervention. The number of outcomes 120-days before and after intervention were fitted with a log linear model, with Poisson distribution. To account for correlation between the repeated measures on patient level, Generalized Estimating Equations (GEE) model was used to estimate the effect. The Difference-in-Difference term was defined as the interaction between the pre-post intervention difference by intervention groups.

Results

Table 4 contains the Difference-in-Difference estimates and 95% CI from the GEE model for the primary outcome. There was an uptrend in PCP visit for both groups, but the CHTP patients had significantly higher increase in PCP visit compared to control group. (DID estimate: 14.4%, 95% CI = 2.5% to 27.6%, p=0.02).

*Table 4: Estimated Difference-in-Difference (DID) with 95% CI, for 120-day pre and post discharge for PCP visit.*

|  | **DID Estimate** | **95% Confidence Interval** | | **p-value** |
| --- | --- | --- | --- | --- |
| **Number of PCP visit** | **1.14** | **(1.02** | **, 1.28)** | **0.02** |
