# Supplementary material for: Implementation of VA care coordination program to improve transitional care for veterans post-non-VA hospital discharge: an incremental cost analysis
Source: Implement Sci Commun. 2023 Nov 13;4:135. doi: 10.1186/s43058-023-00513-4 (PMC10642017; doi:10.1186/s43058-023-00513-4)

**Supplementary Tables: Assessment of Healthcare Cost Differences**

Number of enrolled patients: CHTP pre-matching enrolled 774 patients post-matching included 773 patients – 1546 controls (see supplementary files outlining the matching methods).

**Table 1: VA primary care costs 120 Days before and after index hospitalization**

| **CHTP group** | **Pre/Post Hospitalization**  **(120 days)** | **Median Primary Care Cost** | **Difference in Differences (Median Costs)** |
| --- | --- | --- | --- |
| Intervention | Pre | $ 591.61 |  |
| Intervention | Post | $ 707.92 | $ 116.30 |
| Control | Pre | $ 518.34 |  |
| Control | Post | $ 538.35 | $ 20.01 |

**Table 2: VA outpatient care costs 120 Days before and after index hospitalization**

| **CHTP group** | **Pre/Post Hospitalization**  **(120 days)** | **Median Outpatient Care Cost** | **Difference in Differences (Median Costs)** |
| --- | --- | --- | --- |
| Intervention | Pre | $ 380.21 |  |
| Intervention | Post | $ 543.76 | $ 163.55 |
| Control | Pre | $ 368.20 |  |
| Control | Post | $ 371.75 | $ 3.55 |

**Figure 1: Pre-parallel trends in 120-day non-primary care costs (including zero costs)**


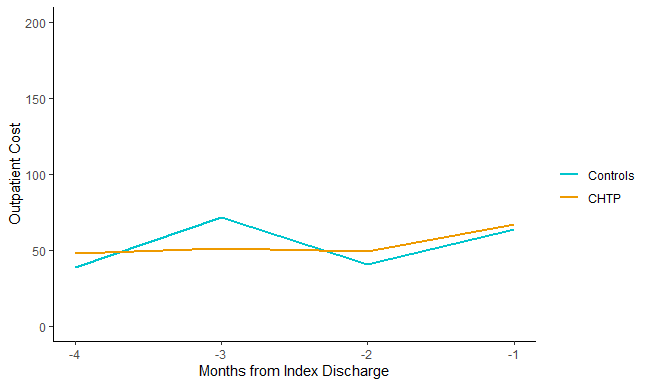


**Figure 2: Pre-parallel trends in 120-day primary care costs (including zero costs)**


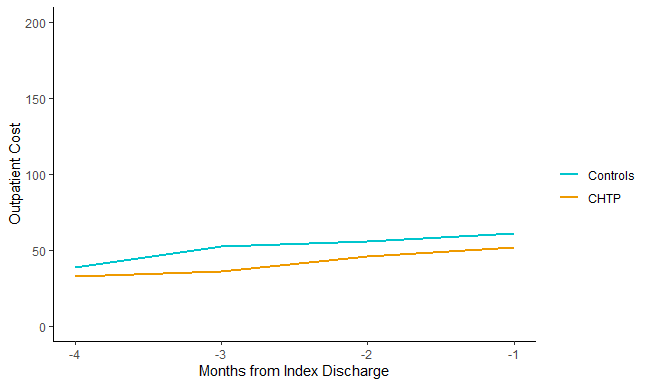

Supplement: Supplementary file 2 — Additional file 2: Supplementary Tables. Assessment of Healthcare Cost Differences. Table 1. VA primary care costs 120 Days before and after index hospitalization. Table 2. VA outpatient care costs 120 Days before and after index hospitalization. Figure 1. Pre-parallel trends in 120-day non-primary care costs (including zero costs). Figure 2. Pre-parallel trends in 120-day primary care costs (including zero costs). [file 43058_2023_513_MOESM2_ESM.docx]
